# Confirmed COVID-19 Cases per Economic Activity during Autumn Wave in Belgium

**DOI:** 10.3390/ijerph182312489

**Published:** 2021-11-27

**Authors:** Johan Verbeeck, Godelieve Vandersmissen, Jannes Peeters, Sofieke Klamer, Sharon Hancart, Tinne Lernout, Mathias Dewatripont, Lode Godderis, Geert Molenberghs

**Affiliations:** 1 Data Science Institute, I-BioStat, Universiteit Hasselt, 3500 Hasselt, Belgium; jannes.peeters@uhasselt.be (J.P.); geert.molenberghs@uhasselt.be (G.M.); 2 IDEWE, External Service for Prevention and Protection at Work, 3001 Heverlee, Belgium; Lieve.Vandersmissen@idewe.be (G.V.); lode.godderis@kuleuven.be (L.G.); 3 Sciensano, Belgian Institute for Health, 1050 Brussels, Belgium; Sofieke.Klamer@sciensano.be (S.K.); sharonhancart@gmail.com (S.H.); Tinne.Lernout@sciensano.be (T.L.); 4 I3h, ECARES and Solvay Brussels School of Economics and Management, Universite Libre de Bruxelles, 1050 Brussels, Belgium; Mathias.Dewatripont@ulb.be; 5 Centre for Environment and Health, Department of Public Health and Primary Care, KU Leuven, 3000 Leuven, Belgium; 6 I-BioStat, Katholieke Universiteit (KU) Leuven, 3000 Leuven, Belgium

**Keywords:** longitudinal study, occupational health, cross-sectional study, Gaussian-Gaussian model, COVID-19, non-pharmaceutical interventions

## Abstract

Some occupational sectors, such as human health and care, food service, cultural and sport activities, have been associated with a higher risk of SARS-CoV-2 infection than other sectors. To curb the spread of SARS-CoV-2, it is preferable to apply targeted non-pharmaceutical interventions on selected economic sectors, rather than a full lockdown. However, the effect of these general and sector-specific interventions on the virus circulation has only been sparsely studied. We assess the COVID-19 incidence under different levels of non-pharmaceutical interventions per economic activity during the autumn 2020 wave in Belgium. The 14-day incidence of confirmed COVID-19 cases per the Statistical Classification of Economic Activities in the European Community (NACE–BEL) sector is modelled by a longitudinal Gaussian-Gaussian two-stage approach. This is based on exhaustive data on all employees in all sectors. In the presence of sanitary protocols and minimal non-pharmaceutical interventions, many sectors with close contact with others show considerably higher COVID-19 14-day incidences than other sectors. The effect of stricter non-pharmaceutical interventions in the general population and non-essential sectors is seen in the timing of the peak incidence and the width and height of the post-peak incidence. In most sectors incidences returned to higher levels after the peak than before and this decrease took longer for the health and care sector. Sanitary protocols for close proximity occupations may be sufficient during periods of low-level virus circulation, but progressively less with increasing circulation. Stricter general and sector-specific non-pharmaceutical interventions adequately decrease COVID-19 incidences, even in close proximity in essential sectors under solely sanitary protocols.

## 1. Introduction

Since the SARS-CoV-2 virus was identified in Wuhan (China) in December 2019, the virus spread globally, causing the largest pandemic in a century. Managing this pandemic has been a challenge worldwide, with non-pharmaceutical interventions (NPIs) to limit virus spread in private life and at work. In Italy it was estimated that, up to May 2020, COVID-19 was probably contracted at work in 30% of cases at working age [1]. As little was known on the role of NPIs on the spread of SARS-CoV-2 at the onset, a general lockdown to manage spread and prevent healthcare system collapse was introduced by many governments. Despite adequately controlling viral spread, a full lockdown leads in time to severe economic and well-being issues. Hence, more targeted NPIs are necessary, such as general and economic sector-specific sanitary protocols, restricting activities and re-opening of selected economic sectors. Therefore, it is essential to gather knowledge on the risk of contracting COVID-19 in different economic activities under varying NPIs.

Economic activities where social distancing is challenging have been related to clusters of COVID-19 cases. In Japan, 61 clusters of COVID-19 were tracked to healthcare (30%) and other care facilities (16%), cultural activities (11%), gyms (8%), ceremonies (3%), and transport (2%) [2]. COVID-19 outbreaks have been documented in economic activities of manufacturing, agriculture/forestry/fishing/hunting, and transportation/warehousing in the US [3] and Canada [4], while several sources report COVID-19 outbreaks in poultry, meat and food processing companies [5] and residential care facilities [6,7]. Employees in bars and restaurants have been shown to have increased COVID-19 risk [8,9] or were involved in clusters of COVID-19 [10]. This was confirmed by the European Centre for Disease Control that examined the number of clusters per sector during the first wave in 15 European countries and the United Kingdom [11]. In Norway, the odds of COVID-19 were 1.1–3 times higher during the first wave in nurses, doctors, dentists, physiotherapists and bus, tram, and taxi drivers relative to the general population at working age [8], while the odds did not increase for some contact professions during the second wave. This suggests that taking appropriate measures at work can contain the spread of the virus at the workplace.

Despite the observed association between activities and COVID-19, this does not provide information on the effect of NPIs on the spread of SARS-CoV-2 in the general and sector-specific population. In some US states, closing and re-opening of bars, restaurant and schools and wearing masks were found to have a significant effect on the spread of the virus, hospitalizations, and deaths [12,13]. In a study combining mobility data with confirmed COVID-19 cases, restaurants, gyms, hotels, cafes, and religious organizations were identified to produce the largest increase in infections when re-opened under pre-pandemic conditions [14]. Therefore, many policy makers have decided to allow re-opening of these locations only with reduced visitors and visiting times to control SARS-CoV-2 spread. To our knowledge, only one study has investigated different NPI strategies. In selected municipalities in Norway, bartenders and waiters had similar rates of COVID-19 in areas with full and partial bans on serving [9].

By linking all COVID-19 confirmed cases of employees with the Statistical Classification of Economic Activities in the European Community (NACE–BEL) code [15] of the main economic activity of their employer, we examine COVID-19 incidence in Belgium under varying non-pharmaceutical interventions during the 2020 autumn wave (Table 1) [16]. We contrast a cross-sectional analysis of the COVID-19 14-day incidence immediately prior to the implementation of more strict measures on 19 October 2020 with a longitudinal analysis of the incidences over the entire autumn, to examine the impact of NPIs. Finally, we evaluate longitudinally high-risk contacts reported by confirmed COVID-19 cases during contact tracing as an alternative way of measuring the effect of interventions.

## 2. Materials and Methods

The Belgian health institute, Sciensano, registers on a daily basis all confirmed COVID-19 cases [17] and forwards them to the National Social Security Office (NSSO), which in turns links these to the Dimona database of active employees. This database covers most employees, such as employees in private and public sectors, interim employment and student jobs, but does not include self-employed or foreign workers (~19% of the working population) as they are not subjected to the Belgian social security scheme.

The daily cases are aggregated at NSSO to weekly incidences (number of cases per 100,000) by NACE-BEL code. NACE-BEL classifies workplaces into 21 main economic sectors (level 1) and then further into ever finer subcategories (levels 2–5), with 943 subcategories at level 5 [15]. Although some companies may be active in more than one sector, only the main NACE-BEL code is assigned. This limitation is particularly important for education because a majority of schools provide both primary and secondary education, with all employees categorized as secondary school personnel in such schools.

The weekly incidences from 8 September 2020 to 25 January 2021 are mapped to 14-day incidences, as is common in epidemiology, by joining two consecutive weeks. Adjacent 14-day incidences share an overlapping week. For the 5 NACE-BEL levels, the highest incidences in the two 14-day periods before the stricter NPIs on 19 October 2020 (29 September–12 October 2020; 6–19 October 2020) are presented, together with the 14-day incidence over all work sectors (~4.5 million individuals) and in the general population (~11.5 million individuals). These two 14-day periods were chosen because the effect on the incidences after the relaxation of NPIs on 23 September are expected to be noticeable only 4–5 days later and because these periods immediately lead up to the stricter 19 October NPIs.

The 95% confidence interval (CI) for the 14-day incidence is calculated on a logit transformation scale of the proportion, after which it is back-transformed to the original scale. Let *Y_st_* be the random variable representing number of COVID-19 confirmed cases [18] in the 14-day period, *t* for sector *s*, *y_st_* its realized value, and *p_st_* the proportion of COVID-19 infected employees, then *I_st_* = *p_st_* × 100,000 is the *14-day incidence*. The 95% CI of the logit transformation of the proportion ${\hat{\lambda }}_{st}=\log\left(\frac{p_{st}}{1-p_{st}}\right),$ with variance ${\hat{\sigma }}_{st}^{2}=\frac{1}{y_{st}\left(1-p_{st}\right)}$, is ${\hat{\lambda }}_{st}\pm 1.96\;\sqrt{{\hat{\sigma }}_{st}^{2}}$. By back-transforming this CI, the 95% CI of the 14-day incidence is:

$$\frac{e^{{\hat{\lambda }}_{st}\;\pm \;1.96\;\sqrt{{\hat{\sigma }}_{st}^{2}}}}{1+e^{{\hat{\lambda }}_{st}\;\pm \;1.96\;\sqrt{{\hat{\sigma }}_{st}^{2}}}\;}.$$

The longitudinal incidence profiles are modelled in a two-step approach by a function that is able to model a peaked curve, which allows for a different shape before and after the peak. The Gaussian-Gaussian function models such an asymmetric bell-shaped curve with different scale parameters on the left and right side of the common peak. In the first step, parameter estimators of the parameter vector $\theta_{s}=\left(\delta_{s1},\;\delta_{s2},\;\alpha_{s1},\;\alpha_{s2},\;\sigma_{s1},\;\sigma_{s2},\;\sigma_{s}^{2},\;\nu_{s}\right)\;$are obtained per NACE-BEL sector *s* by maximizing the Gaussian likelihood:

$$L_{s}\left(\theta_{s}|I_{s1},\;\ldots ,\;I_{sT}\right)=\overset{T}{\underset{t=1}{\prod }}\frac{1}{\sigma_{s}\sqrt{2\pi }}e^{-\frac{{\left[I_{st}-\mu_{st}\right]}^{2}}{2\sigma_{s}^{2}}}$$

but with a non-linear mean function that is itself (asymmetrically) bell shaped:

$$\mu_{st}=\{\begin{array}{c} \delta_{s1}+\alpha_{s1}e^{-\frac{{\left(t-\nu_{s}\right)}^{2}}{\sigma_{s1}^{2}}}\;\;\;\;\;\;\;if\;t<\nu_{s},\\ \delta_{s2}+\alpha_{s2}e^{-\frac{{\left(t-\nu_{s}\right)}^{2}}{\sigma_{s2}^{2}}}\;\;\;\;\;\;\;if\;t\geq \nu_{s}, \end{array}$$

with $\nu_{s}$ the time at the peak in sector *s*, $\delta_{s1}+\alpha_{s1}\equiv \delta_{s2}+\alpha_{s2}$ the height of the peak, $\delta_{s1}$ and $\delta_{s2}$ the height of the incidence in the plateau phase before and after the peak, respectively, and $\sqrt{2\ln\left(2\right)}\sigma_{s1}\;$ and $\sqrt{2\ln\left(2\right)}\sigma_{s2}\;$the half-width of the peak before and after the peak. For each NACE-BEL code the fully parameterized Gaussian-Gaussian model is fitted. When there is evidence of over-parametrization, parameters left and right of the peak are equally set in steps until convergence. The order in which the parameters are equally set, whenever needed, are the $\alpha_{sj}$, the $\sigma_{sj}$, and the $\delta_{sj}$ (*j* = 1, 2), respectively, until a symmetric Gaussian curve is obtained.

In a second step, the variance–covariance matrix of the parameter estimators is used to construct 95% confidence intervals (CI) for the parameter estimators. Extreme parameter values are identified as those where the CI is excluding the mean of the parameter estimates.

Note that in a fully hierarchical analysis, the vector *θ_s_* would be decomposed in a fixed and random part in a non-linear mixed-effects model. While this would take the correlation between observations in the same sectors into account in the most principled fashion, the two-stage approach is considered an adequate and computationally feasible approximation [19].

Since precision in small NACE-BEL sectors is low, we only analyzed sectors at levels 1, 2, and 3, with a minimum of 10,000 employees. For levels 4 and 5, the minimum was set at 3000 and 1500, respectively.

Cross-sectional data are partially included in supplementary Tables S1–S5. All NACE-BEL sectors with their corresponding 14-day incidence and confidence interval, as well as longitudinal data are available upon reasonable request. Various rankings or aggregates of sectors can easily be constructed from this information.

Data on workplace high-risk contacts of confirmed COVID-19 cases are available from the IDEWE contact tracing database. IDEWE is one of the largest occupational health services in Belgium and responsible for the well-being of approximately 800,000 employees from 33,000 companies or institutions, covering more than 20% of Belgian workers. IDEWE is active in all economic sectors, with a predominance in healthcare. More than 20% of all employees under medical surveillance of IDEWE are working in the healthcare sector.

Since 29 October 2020, the COVID-Contact Tracing application, developed by IDEWE for its employers, registers, in a standardized fashion, information on COVID-19 incidences and on high- and low-risk contacts of index cases. Most of the index cases are employees, but also seasonal workers, interns, pupils, and external people working at company and institutional premises that tested positive are contacted. Contact tracing of high- and low-risk contacts, defined according to Sciensano guidelines [18], within the company is performed and measures taken for within-company high-risk contacts (testing, 7- or 10-days quarantine). Contacts exterior to the company are identified and followed-up through the regular contact tracing.

Employers in the IDEWE system are grouped by customer segment in one of nine regional offices, named after the city where they are located. Most Belgian provinces have one regional office, except Namur that serves all of Wallonia and Antwerp that is served by the regions Antwerpen, Mechelen, and Turnhout. IDEWE distinguishes between ten customer segments based on NACE codes, but an exact link with the NSSO codes is not fully possible.

Some larger IDEWE companies have organized contact tracing via their internal prevention service which is not included in this analysis and potentially leading to underestimation of index cases. For some segments this underestimation might be more important. Under-reporting may also arise because the tracing application reports zero high-risk contacts for an index case by default, which might be incorrect for an index that is non-contactable or refuses to respond. On the other hand, over-reporting might occur in the education segment. The contact tracing for schools is performed by student guidance centers (SGC), who forward the contract tracing of pupils to IDEWE if employees might be involved as high- or low- risk contact. The SGC tracing is center dependent and often only index cases with high-risk contacts are forwarded to IDEWE. The number of index cases for the segments ‘construction’, ’emergency services’, and ‘agriculture’ and for the region ‘Namur’, were very low during some time periods, leading to imprecise estimates.

For the index cases that were reported via IDEWE contact tracing, the mean number of high-risk contacts and the four-weekly percentage of index cases with two or more high-risk contacts are described per work segment and per region.

This study was conducted in accordance with Belgian and international legislation guidelines on personal data handling to guarantee privacy. In this context, the approval of the local ethics committee OG 117 for data collection during occupational health surveillance was obtained. During medical examination, every worker is informed about the retrospective analysis of anonymized data gathered during the examination with the possibility of opting out.

## 3. Results

**Figure 1:**
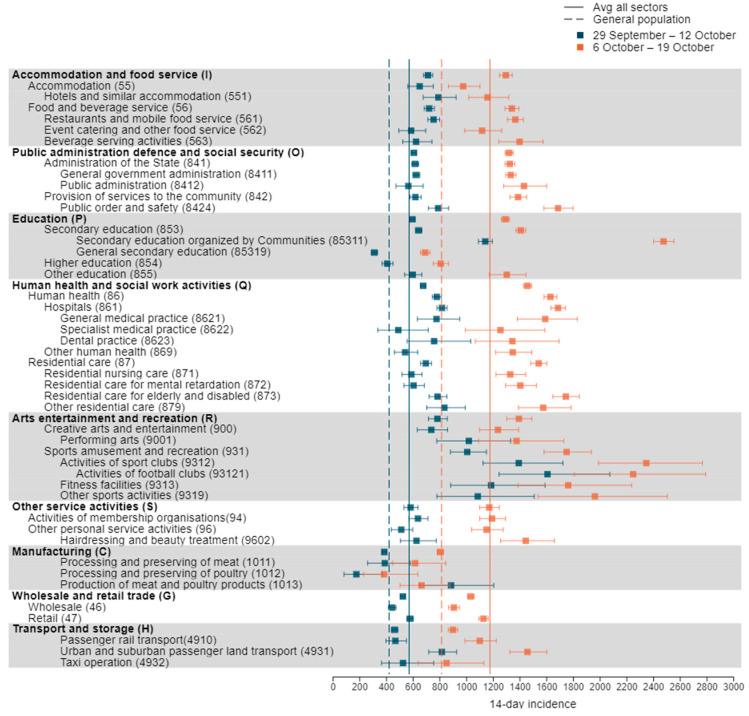
The 14-day incidence of COVID-19 in selected sectors in periods 29 September–12 October and 6–19 October.

At NACE-BEL level 1, the following sectors with a minimum of 10,000 employees show a 14-day incidence above the NACE-BEL average in both minimal NPI periods before 19 October 2020: arts, entertainment and recreation; accommodation and food service activities; human health and social work activities; public administration and defence; and education (Figure 1 and Table S1). In each of these sectors, the subsectors responsible for the increased incidence are identified.

As the human health and social work sector is subject to frequent close contacts, it is no surprise that its incidence is among the highest among the lowermost level 3–5 sectors (Figure 1 and Tables S2–S5). However, in other sectors, even higher incidences are reported. For example, in the arts, entertainment and recreation sector, high incidences occur mainly from activities of sport clubs (especially football clubs), fitness activities, and other sport activities (Figure 1 and Tables S4 and S5). While in the education sector, education organized by regional authorities (sector 85,311) exhibit a higher incidence than the care sector (Figure 1 and Tables S3 and S5).

The accommodation and food service incidence is of a comparably high level as that of the health and care sectors and is similar between hotels, restaurants, and bars between 29 September–12 October (Figure 1, Tables S1–S5). Within public administration, defence and social security, the incidence in governmental and general administration services and the public order and safety sector exceeds the all-sector average and is comparable to the incidences in the health and care sectors (Figure 1).

Although the 14-day incidence in other service activities and transportation and storage at NACE-BEL level 1 is around the all-sector average, individual subsectors show an increased incidence. The incidences in the non-medical contact professions and urban and suburban passenger surface transportation stand out with incidences similar to the health and care sector (Figure 1).

Although identified in the literature as high-risk sectors, the spread of COVID-19 in meat and poultry processing sectors (sector 1011, 1012, 1013) seems well controlled in the periods immediately prior to the October measures, with an incidence slightly lower than in the general population (Figure 1). Likewise, the incidences in wholesale and retail trade are well controlled before 19 October 2020, although some sectors show an elevated incidence (sectors 4729, 4631, 4764, 4773) (Figure 1).

**Figure 2:**
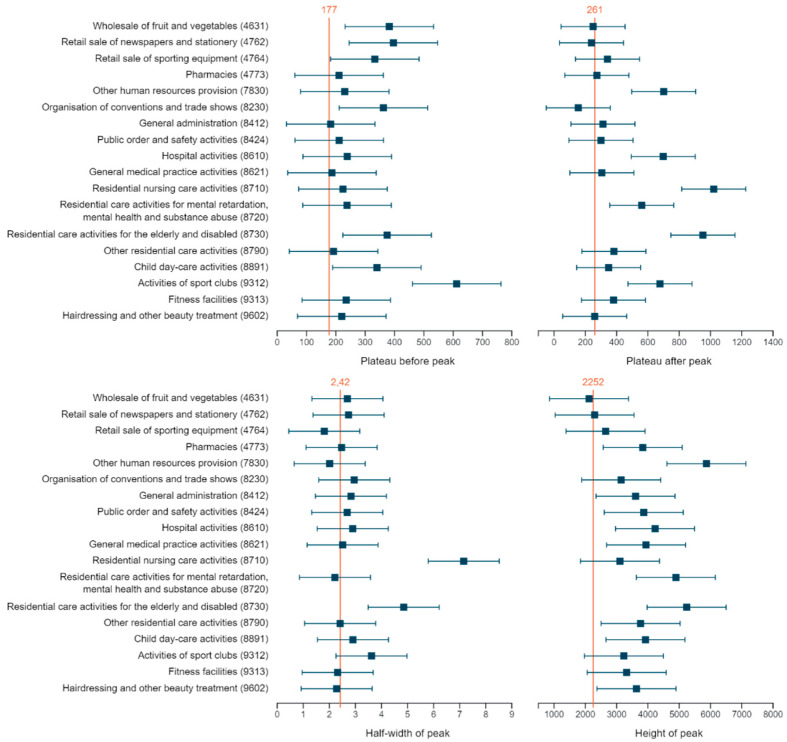
Forest plots of characteristics of the longitudinal profile of selected sectors. The plateaus before and after the peak are related to the 14-day incidence. The height of the peak is the 14-day incidence at the highest moment in the curve and the half-width of the peak is the number of weeks it takes for the curve to reduce the 14-day incidence by a half.

The minimal NPI period before 19 October can further be studied via the longitudinal profile, which is represented by the pre-peak plateau in the Gaussian-Gaussian model. From the modelling we learn that the incidences before the peak in sports activities, more specifically activities of sport clubs and residential care for elderly and disabled, were significantly elevated (Figure 2). At level 4, additionally, child day care, organisation of conventions and trade shows, and some wholesale and retail trade sectors have an increased incidence before the peak (Figure 2).

**Figure 3:**
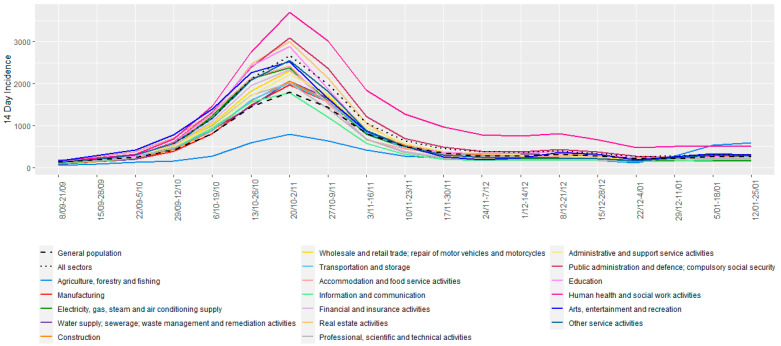
The 14-day incidence of COVID-19 in sectors with a minimum of 10,000 employees at level 1.

Both the cross-sectional and longitudinal analyses show that for the majority of sectors, the autumn wave reaches its peak incidence around 20 October–2 November 2020 (Figure 3), which is significantly higher for human health and residential care activities (Figure 2 and Figure 3). Within human health activities, both hospitals and general medical practice have a higher incidence, while for residential care activities, most sectors show an extreme peak (sectors 872, 873, 879) (Figure 2). At level 4, additionally fitness centre activities, non-medical contact professions, general administration, federal and local police, pharmacies, and other human resource provision have a significantly elevated peak incidence (Figure 2).

**Figure 4:**
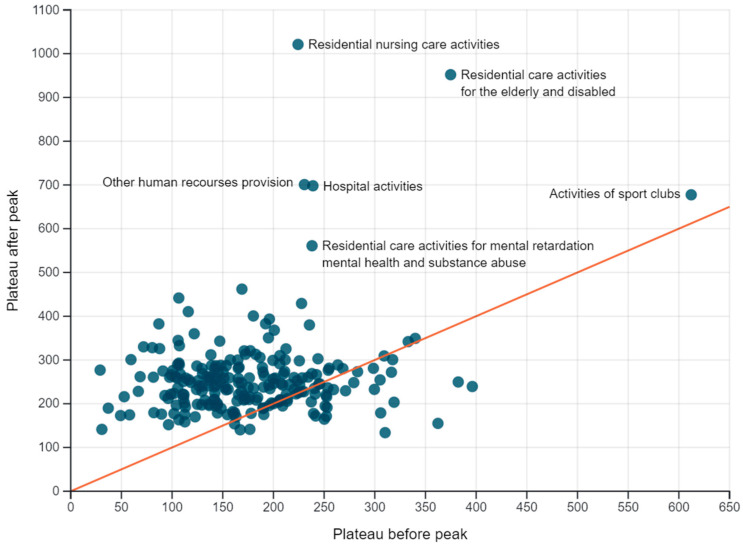
Comparison of the plateau before and after the peak in sectors at level 4.

Two parameters in the longitudinal analysis inform us about the effect of the stricter NPIs of 19 October 2020. The plateau after the peak captures the incidence of which the sector decreased, while the half-width quantifies the speed of decrease. Not surprisingly, human health and residential care activities have a significantly higher post-peak incidence level compared to the other sectors, while the latter sector also has a larger half-width (Figure 2 and Figure 3). Within human health activities, hospitals have a higher post-peak incidence, while for the residential care activities most sectors show both an increased incidence after the peak (sector 871, 872, 873), and a longer half-width (sector 871, 873). At level 4, activities of sports clubs, and other human resource provision (sector 7830), had a significantly elevated post-peak incidence (Figure 2). Of note, for most sectors the post-peak plateau is higher than the pre-peak one (Figure 4).

**Figure 5:**
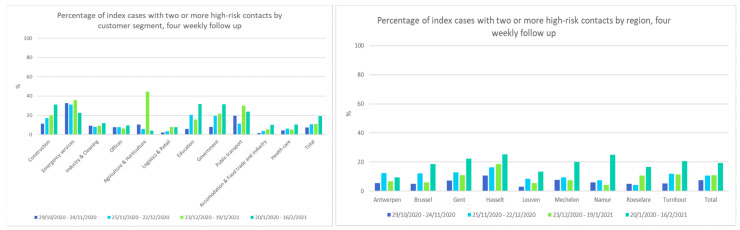
Four-weekly percentage of index cases with two or more high-risk contacts by segments under surveillance (**left**) and by geographical region (**right**).

In the contact tracing from IDEWE, the high-risk contacts of 6016 index cases were recorded between 29 October 2020 and 18 February 2021. The number of high-risk contacts per index case varied from 0 to 56, with more than 99% having less than 10 high-risk contacts. For 5952 index cases, the customer segment and region were known. The frequency of index cases that reported two or more high-risk contacts increased over time in most segments and all regions (Figure 5). Index cases in the segments emergency services, education, government, and public transport reported a higher mean number of high-risk contacts over this period, compared to other segments, while healthcare and accommodation and food trade and industry index cases reported relatively low values (Figure S1).

## 4. Discussion

When studying the 14-day incidence of COVID-19 per economic activity in Belgium, we have found that, despite sanitary protocols, the risk of COVID-19 infection was increased in many sectors where close and/or prolonged contact with others is high. Stricter NPIs in non-essential sectors adequately decrease COVID-19 incidences, even in those sectors with frequent close contact that remain open. Although, in most sectors incidences returned to higher levels after the peak than before. Interestingly, the decrease in the health and care sector took longer. Finally, the increasing amount of reported high-risk contacts by COVID-19 confirmed cases during contact tracing hints towards a decreasing motivation over time to adhere to NPIs. The observed pattern of reporting high-risk contacts coincides with the varying motivation to adhere to the NPIs observed in the Belgian Great Corona Study [20].

The strengths of our analysis are that the data include all confirmed COVID-19 cases among all employees, interim employment, and students with jobs, across all economic activities. It is thus complete and enjoys high precision, unlike when based on a random sample in a restricted set of occupations or a self-completed sample survey.

The absence of information on COVID-19 incidence in self-employed workers is a limitation. As the proportion of self-employed workers per NACE-BEL sector is variable, this might have a variable impact. Additionally, the data depend on the COVID-19 testing strategy, which changed on 20 October 2020 when testing of asymptomatic individuals following a high-risk contact was suspended until 23 November 2020 to safeguard testing laboratory capacity. Arguably, this likely impacts most sectors equally. Finally, NACE-BEL codes are assigned only to the main activity of a company and no inference can be made regarding the location of infection (workplace or elsewhere) nor the location of employment (work, telework, temporarily unemployed). The results, however, do reflect the behaviour and potential risk of spreading COVID-19 by employees in a sector.

Economic activities with increased incidence are mostly sectors with the professional need for close proximity to other people. The risk inflation factor Jobs At Risk Index (JARI) [21] indeed suggests that a higher COVID-19 incidence is expected in occupations where employees are in close contact with others and/or with infections, such as in healthcare activities, residential care, prison staff and undertakers. However, in Belgium, sectors that are labelled by JARI as occupations with only close proximity and no regular contact with disease (education, law enforcement, fitness, beauty, retail, musicians/actors, restaurants and bars, and transport) have an equally high or further elevated incidence. Since index cases in the healthcare segment report a relatively small amount of high-risk contacts, this suggests that NPIs in healthcare are efficient and/or healthcare employees are effective in avoiding high-risk contacts. On the contrary, the NPIs prior to the autumn wave (Table 1) may have been insufficient for close proximity occupations. Arguably, employees’ behaviour in these sectors could be more risky (on the work floor and/or beyond), as evidenced by increased reporting of high-risk contacts in public transport for example. Sanitary protocols may be sufficient during periods of low-level virus circulation but progressively less with increasing incidence.

Our results contrast with the findings of no increased incidence in sports activities of the SafeActive survey on self-reported COVID-19 in a sample of fitness and exercise facilities [22] and that of no difference in incidence in a sample of occupations in a UK survey [23]. Our findings agree with the analysis of mobility data, identifying gyms, bars, and restaurants as high-risk locations of infection [14], and the reports of clusters of COVID-19 and outbreaks [2,3,4,5,6,7,8,9,10]. Various high-incidence sectors are mentioned as potential locations of infection by index patients during contact tracing. Places mentioned most as activity or event visited two weeks before infection are restaurants and bars, sports activities, public activities, wellness and hairdressers and fitness facilities (Flemish contact tracing). While not formal proof for the place of infection, the increased incidence in these sectors is striking.

The effect of the stricter NPIs taken on 19 October 2020 is seen in the timing of the peak incidence, which for most sectors, including sectors with restricted activity and unrestricted sectors, is in the 2-week period past this date. Despite their forefront position [24], employees in the food industry seem adequately protected and well informed on protective measures in Belgium, as incidences in food retail decreased to the all-sector average after the peak and the number of high-risk contacts reported by accommodation and food trade and industry are low.

The effect of the NPIs is also seen in the width and height of the post-peak incidence. Unsurprisingly, the peak width is significantly broader for human health activities and residential care activities, contradicting that infection preventive protocols in healthcare are adequate to prevent SARS-CoV-2 infections [25]. For most activities, post-peak incidence is higher than pre-peak, reflecting controlled but increased SARS-CoV-2 circulation. Human health activities, residential care activities, activities of sports clubs and other human resource provisions have a significantly higher post-peak incidence. Although incidence in sports activities is largely influenced by virtually unrestricted activities of professional football clubs [26], fitness facilities, other sports activities, and activities of leagues and sports federations also have an increased peak and/or pre-peak incidence.

The success of the NPIs to curb the second wave notwithstanding, the increasing number of index cases over time reporting two or more high-risk contacts potentially demonstrates the decreasing motivation to adhere to these measures [20]. Evidently, increasing high-risk behaviour by a part of the general population may result in delays towards relieving NPIs by the decision makers.

Further insights on the NPIs should be gained from, including information on self-employed workers.

## 5. Conclusions

Despite the sanitary protocols present, employees in activities where close and/or prolonged contact with others is high, exhibit increased risk of COVID-19 infection, even higher than the high-risk sector of human health and care. NPIs in the general population and non-essential sectors adequately decreases COVID-19 incidences, even in those sectors with frequent close contacts under solely sanitary protocols, for example, human health and care activities and food shops. Although the measures are adequate, the increasing amount of reported high-risk contacts by COVID-19 confirmed cases during contact tracing hints towards a decreasing motivation over time to adhere to the measures.

These insights offer guidance to policy makers on which economic activity to restrict or subject to stricter protocols to better control COVID-19 endemics or a future pandemic, whilst keeping the work floor as safe as possible. Indeed, better knowledge of occupational risks enables the development of both improved and more targeted risk mitigation measures to prevent the spread of the virus and informed measures to be taken in the event of another future pandemic. For example, in Belgium, our analyses have contributed to policy decisions about temporary closures of sectors (bars and restaurant, non-medical contact professions, etc.) whilst keeping others open (e.g., non-essential shops). These analyses are indispensable for making informed decisions about exit measures and a ‘new normal’ way of working. In addition, improved evidence has the ability to inform any decisions on the recognition of COVID-19 as an occupational illness and which occupations or occupational circumstances could be eligible for compensation.

## Figures and Tables

**Table 1:** Overview of non-pharmaceutical interventions in Belgium.

Date	Description
18 March 2020	Closing schools and suspending all cultural, leisure, and non-essential activities
Summer 2020	Resume most activities with strict protocols, including restrictions on capacity, dwelling time, and social contacts
1 September 2020	Wearing a mask is mandatory in public places Teleworking (working from home) is recommended Social bubble of close contacts consists of five people per month Shopping is possible as a couple, with no time limit, but with a maximum capacity applicable to the shop Restaurants and bars are open, with minimal distance between tables of 1.5 m and sanitary protocols Sport facilities are open with restrictions on capacity and with sanitary protocols Primary schools are open without restrictions. Secondary schools are open with face mask mandate for pupils and teachers. Higher education is open with restrictions on capacity per lecture hall and mask mandate for students and teachers; Public events are allowed with a maximum of 200 people indoors and 400 outdoors
23 September 2020	Shopping without maximum capacity allowed and the social bubble of five contacts replaced to unlimited contacts, but a maximum of 10 adults per social gathering
19 October 2020	Close contacts are limited to one person Private and public gatherings are limited to four people Teleworking is mandatory for all occupations where this is possible Bars and restaurants are closed A curfew is installed between midnight (earlier in some regions) and 5 a.m., with a ban on alcohol sales from 8 p.m. onward Indoor activities can continue under existing protocols Audiences for sports events are halved from 400 to 200 spectators
23 October 2020	Audiences banned for sports events, class occupancy rate reduced in higher education, the capacity of indoor cultural events reduced and amusement parks and zoological gardens closed
2 November 2020	Non-essential shops and non-medical contact professions closed. Hotels remained open, but bars and restaurants were closed

## Data Availability

Cross-sectional data are included in the article or uploaded as supplementary information. Longitudinal data are available upon reasonable request.

## Supplementary Materials

The following are available online at https://www.mdpi.com/article/10.3390/ijerph182312489/s1, Table S1: 14-day incidence of COVID-19 in sectors with minimum 10,000 employees at level 1 on both periods prior to 19 October 2020 non-pharmaceutical interventions, Table S2: 14-day incidence of COVID-19 of 10 sectors with the highest incidence at level 2 on both periods prior to 19 October 2020 non-pharmaceutical interventions, Table S3: 14-day incidence of COVID-19 in sectors with the highest incidence at level 3 on both periods prior to 19 October 2020 non-pharmaceutical interventions, Table S4: 14-day incidence of COVID-19 in sectors with the highest incidence at level 4 on both periods prior to 19 October 2020 non-pharmaceutical interventions, Table S5: 14-day incidence of COVID-19 in sectors with the highest incidence at level 5 on both periods prior to 19 October 2020 non-pharmaceutical interventions, Figure S1: The mean number of high-risk contacts per index case by segments under surveillance.
