# Supplementary material for: Confirmed COVID-19 Cases per Economic Activity during Autumn Wave in Belgium
Source: Int J Environ Res Public Health. 2021 Nov 27;18(23):12489. doi: 10.3390/ijerph182312489 (PMC8656663; doi:10.3390/ijerph182312489)
Supplement: Supplementary file 1 [file ijerph-18-12489-s001.zip › ijerph-1447404-supplementary.pdf]

Supplementary Material to Confirmed COVID-19 cases per economic activity during Autumn wave in Belgium

Supplementary Tables

Table S1. 14-Day incidence of COVID-19 in sectors with minimum 10,000 employees at Level 1 on both periods prior to the 19th October non-pharmaceutical interventions

29 September–12 October 2020			
DESCRIPTION	NACE Code	Employees	Incidence (95%CI)
Arts, entertainment and recreation	R	60382	785(718;859)
Accommodation and food service activities	I	219468	714(680;750)
Human health and social work activities	Q	588348	678(657;699)
Public administration and defence; compulsory social security	O	542270	608(588;629)
Education	P	531993	597(577;618)
Other service activities	S	81336	584(534;639)
**All sectors**		**4390750**	**573(566;580)**
Real estate activities	L	24599	561(475;662)
Financial and insurance activities	K	125758	528(489;570)
Wholesale and retail trade; repair of motor vehicles and motorcycles	G	634221	526(508;544)
Administrative and support service activities	N	350493	507(484;531)
Electricity, gas, steam and air conditioning supply	D	21032	504(417;609)
Information and communication	J	124635	479(442;519)
Professional, scientific and technical activities	M	199790	476(447;507)
Transportation and storage	H	272414	464(439;490)
Water supply; sewerage; waste management and remediation activities	E	35281	445(381;520)
Construction	F	226328	433(407;461)
**General population**			**423**
Manufacturing	C	542636	387(371;404)
Agriculture, forestry and fishing	A	40373	161(126;205)
6–19 October 2020			
DESCRIPTION	NACE Code	Employees	Incidence (95%CI)
Human health and social work activities	Q	587069	1358(1329;1388)
Arts, entertainment and recreation	R	62169	1266(1181;1357)

Public administration and defence; compulsory social security	O	556718	1206(1177;1235)
Real estate activities	L	24568	1205(1076;1349)
Accommodation and food service activities	I	223310	1202(1158;1248)
Education	P	542963	1193(1164;1222)
**All sectors**		**4390832**	**1178(1168;1188)**
Electricity, gas, steam and air conditioning supply	D	21026	1122(989;1274)
Other service activities	S	81781	1103(1034;1177)
Financial and insurance activities	K	124922	1058(1003;1117)
Wholesale and retail trade; repair of motor vehicles and motorcycles	G	638003	965(942;990)
Professional, scientific and technical activities	M	198992	926(885;969)
Administrative and support service activities	N	354068	913(882;944)
Information and communication	J	125087	909(858;963)
Water supply; sewerage; waste management and remediation activities	E	35462	863(772;965)
Transportation and storage	H	274308	847(814;882)
Construction	F	226163	821(784;859)
**General population**			**816**
Manufacturing	C	547403	741(719;764)
Agriculture, forestry and fishing	A	40099	217(176;268)

Table S2. 14-Day incidence of COVID-19 of 10 sectors with the highest incidence at Level 2 on both periods prior to the 19th October non-pharmaceutical interventions

29 September–12 October 2020			
DESCRIPTION	NACE Code	Employees	Incidence (95%CI)
Sports activities, amusement and recreation activities	93	26911	955(846;1078)
Human health activities	86	261154	780(747;814)
Creative, arts and entertainment activities	90	21922	739(634;861)
Food and beverage service activities	56	191978	723(686;762)
Residential care activities	87	162464	698(659;740)
Accommodation	55	27454	652(563;754)
Activities of membership organisations	94	47887	639(571;714)
Activities auxiliary to financial services and insurance activities	66	30351	626(543;721)
Security and investigation activities	80	20096	622(522;741)
Office administrative, office support and other business support activities	82	36731	618(543;704)
**All sectors**		**4390750**	**573(566;580)**
**General population**			**423**
6–19 October 2020			
DESCRIPTION	NACE Code	Employees	Incidence (95%CI)
Sports activities, amusement and recreation activities	93	26687	1660(1513;1820)

Human health activities	86	261680	1631(1583;1680)
Residential care activities	87	162476	1543(1484;1604)
Food and beverage service activities	56	189940	1342(1291;1395)
Public administration and defence; compulsory social security	84	542998	1321(1291;1352)
Security and investigation activities	80	20015	1304(1156;1471)
Education	85	534158	1294(1264;1325)
Activities auxiliary to financial services insurance activities	66 and	30322	1273(1153;1406)
Real estate activities	68	24582	1257(1125;1404)
Office administrative, office support and business support activities	82 other	37037	1242(1134;1360)
**All sectors**		**4390832**	**1178(1168;1188)**
**General population**			**816**

Table S3. 14-Day incidence of COVID-19 in sectors with the highest incidence at Level 3 on both periods prior to the 19th October non-pharmaceutical interventions

29 September–12 October 2020			
DESCRIPTION	NACE Code	Employees	Incidence (95%CI)
Sports activities	931	21131	1008(882;1152)
Other residential care activities	879	15412	837(705;994)
Hospital activities	861	210745	819(781;858)
Hotels and similar accommodation	551	20076	792(678;925)
Residential care activities for the elderly and disabled	873	66667	786(722;856)
Restaurants and mobile food service activities	561	149471	756(713;801)
Activities of call centres	822	10133	750(599;938)
Creative, arts and entertainment activities	900	21922	739(634;861)
Other passenger land transport	493	40751	719(641;806)
Medical and dental practice activities	862	22695	705(604;823)
**All sectors**		**4390750**	**573(566;580)**
**General population**			**423**
6–19 October 2020			
DESCRIPTION	NACE Code	Employees	Incidence (95%CI)
Sports activities	931	21062	1752(1583;1938)
Residential care activities for the elderly and disabled	873	66609	1746(1649;1848)
Hospital activities	861	210960	1688(1634;1744)
Other residential care activities	879	15472	1577(1392;1786)
Medical and dental practice activities	862	22765	1454(1306;1618)
Office administrative and support activities	821	11777	1435(1235;1666)
Secondary education	853	404968	1409(1373;1446)

Residential care activities for mental retardation, mental health and substance abuse	872	39972	1406(1295;1526)
Beverage serving activities	563	19500	1400(1244;1575)
Activities of call centres	822	10367	1389(1181;1633)
**All sectors**		**4390832**	**1178(1168;1188)**
**General population**			**816**

Table S4. 14-Day incidence of COVID-19 in sectors with the highest incidence at Level 4 on both periods prior to the 19th October non-pharmaceutical interventions

29 September–12 October 2020			
DESCRIPTION	NACE code	Employees	Incidence (95%CI)
Activities of sports clubs	9312	5954	1394(1126;1725)
Other human resources provision	7830	4320	1250(959;1629)
Fitness facilities	9313	3707	1187(884;1591)
Other retail sale of food in specialised stores	4729	3312	1117(810;1538)
Other sports activities	9319	3220	1087(781;1510)
Performing arts	9001	5196	1020(780;1333)
Other amusement and recreation activities	9329	3398	883(618;1260)
Manufacture of air and spacecraft and related machinery	3030	5257	875(656;1166)
Other residential care activities	8790	15412	837(705;994)
Service activities incidental to air transportation	5223	5968	821(621;1085)
**All sectors**		**4390750**	**573(566;580)**
**General population**			**423**
6–19 October 2020			
DESCRIPTION	NACE code	Employees	Incidence (95%CI)
Other human resources provision	7830	4326	2381(1967;2880)
Activities of sports clubs	9312	5875	2349(1991;2769)
Other sports activities	9319	3208	1964(1537;2506)
Child day-care activities	8891	25658	1824(1667;1995)
Other retail sale of food in specialised stores	4729	3305	1785(1385;2297)
Fitness facilities	9313	3741	1764(1388;2239)
Residential care activities for the elderly and disabled	8730	66609	1746(1649;1848)
Other credit granting	6492	3230	1734(1337;2247)
Manufacture of air and spacecraft and related machinery	3030	5251	1714(1396;2103)
Public order and safety activities	8424	53021	1688(1582;1801)
**All sectors**		**4390832**	**1178(1168;1188)**
**General population**			**816**

Table S5. 14-Day incidence of COVID-19 in sectors with the highest incidence at Level 5 on both periods prior to the 19th October non-pharmaceutical interventions

29 September–12 October 2020			
DESCRIPTION	NACE Code	Employees	Incidence (95%CI)
Activities of football clubs	93121	3605	1609(1246;2076)
Activities of leagues and sports federations	93191	1961	1428(988;2060)
Other human resources provision	78300	4320	1250(959;1629)
Fitness facilities	93130	3707	1187(884;1591)
Other retail trade of food in specialised stores	47299	2857	1155(822;1620)
General ordinary secondary education	85311	159055	1143(1092;1196)
General construction of office buildings	41202	1756	1139(736;1759)
General social services with accommodation	87902	2500	1080(742;1570)
Production of shows by artistic ensembles	90012	4924	1056(806;1383)
Other cleaning activities	81290	2846	1019(709;1463)
**All sectors**		**4390750**	**573(566;580)**
**General population**			**423**
6–19 October 2020			
DESCRIPTION	NACE Code	Employees	Incidence (95%CI)
Manufacture of weapons and ammunition	25400	2183	2657(2060;3422)
General construction of office buildings	41202	1761	2556(1914;3406)
General ordinary secondary education	85311	159806	2478(2403;2555)
Other human resources provision	78300	4326	2381(1967;2880)
Activities of football clubs	93121	3556	2250(1811;2793)
Activities of leagues and sports federations	93191	1956	2250(1678;3010)
Distribution of gaseous fuels through mains	35220	1881	2126(1563;2886)
Activities of medical laboratories	86901	5159	1977(1631;2395)
Nurseries and day-care centres	88911	22564	1950(1778;2139)
Motion picture projection activities	59140	1633	1837(1287;2615)
**All sectors**		**4390832**	**1178(1168;1188)**
**General population**			**816**

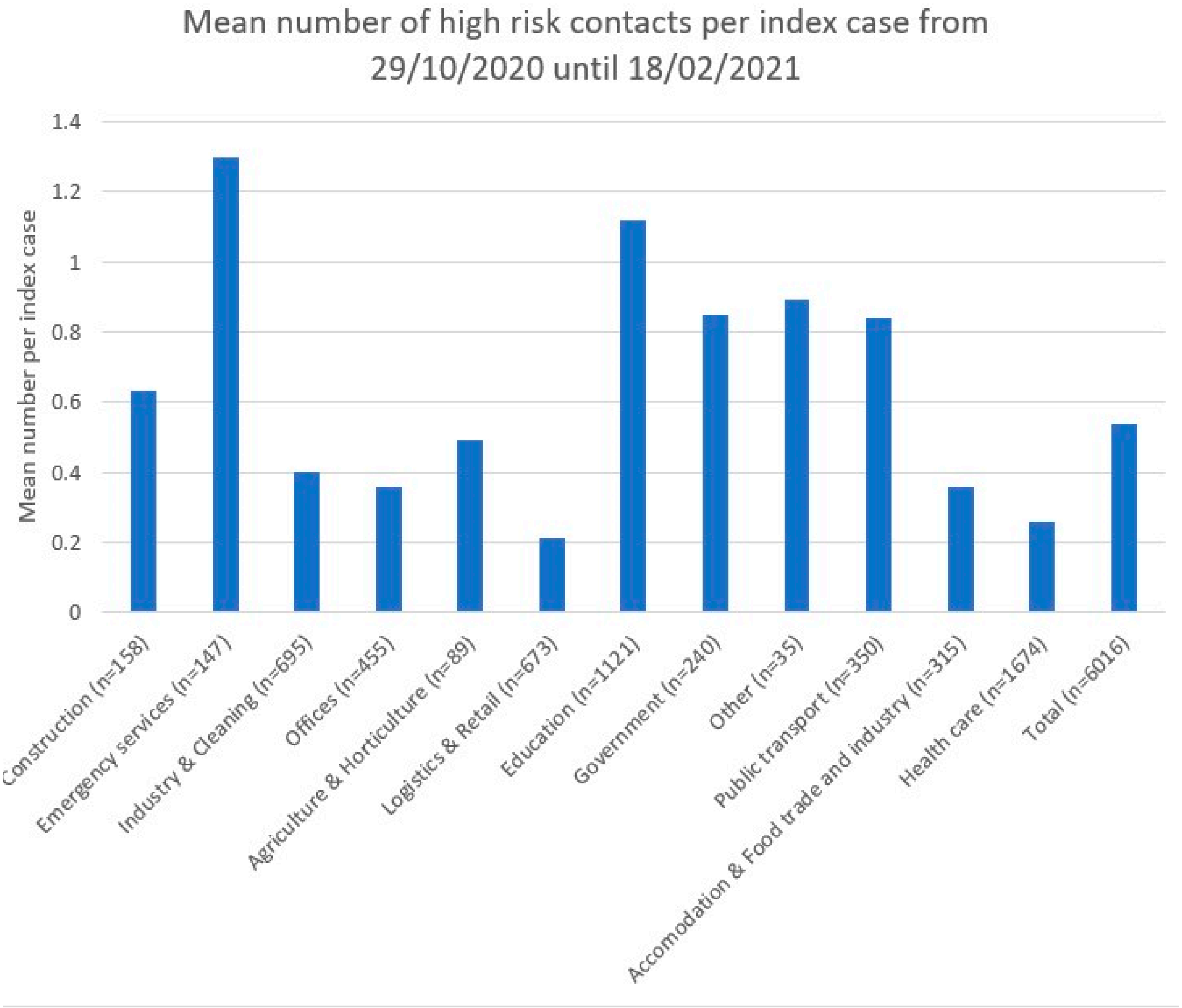

Figure S1. The mean number of high-risk contacts per index case by segments under surveillance
